# Supplementary material for: True oxygen reduction capacity during photosynthetic electron transfer in thylakoids and intact leaves
Source: Plant Physiol. 2022 Feb 15;189(1):112–28. doi: 10.1093/plphys/kiac058 (PMC9070831; doi:10.1093/plphys/kiac058)
Supplement: kiac058_Supplementary_Data [file kiac058_supplementary_data.pdf]

Supplemental Material:

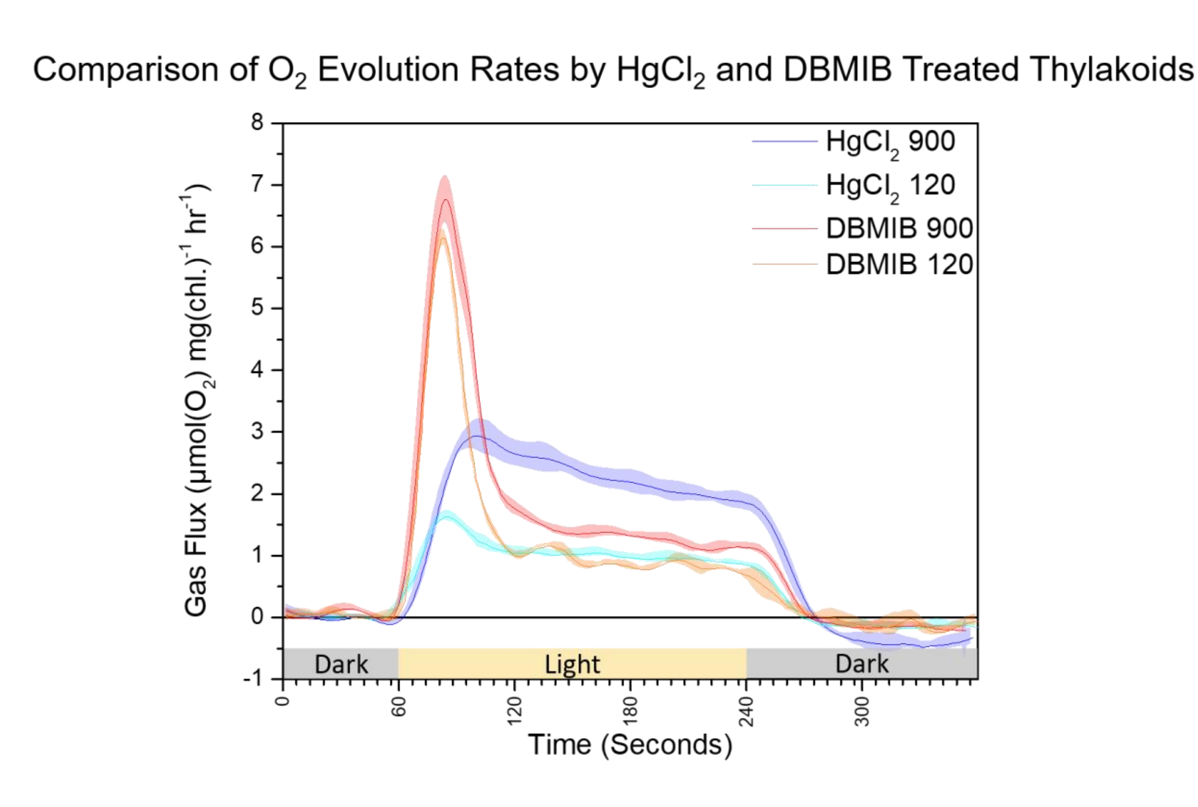

Supplemental Figure S1 Direct comparison of O_2 Evolution rates by thylakoid membranes incubated with DBMIB (10 μM) and $HgCl_2$ (2 mg mL^{-1}) at 120 and 900 μmol photons m^{-2} s^{-1}. The figure compiles the O_2 evolution data from primary figures 3 and 4 to highlight (1) HgCl2 was no more toxic to PSII then incubation with DBMIB. (2) At low irradiance very little difference was observed between inhibitor treatments, although being redox inert $HgCl_2$ lacks the capacity to accept electrons from PSII during the initial period of illumination. (3) The difference in rates between low and high light was much larger in $HgCl_2$ samples then DBMIB samples – potentially suggesting that the capacity for the PQ pool (or potentially PTOX), to reduce O_2 is more limited then including involvement of Cyt-b_6f complex. Recall from main figs (Fig. 3 and Fig. 4) and that neither pathway supported the accumulation of H_2O_2, suggesting any superoxide formed under these conditions must have been rapidly quenched back to O_2 and H_2O.

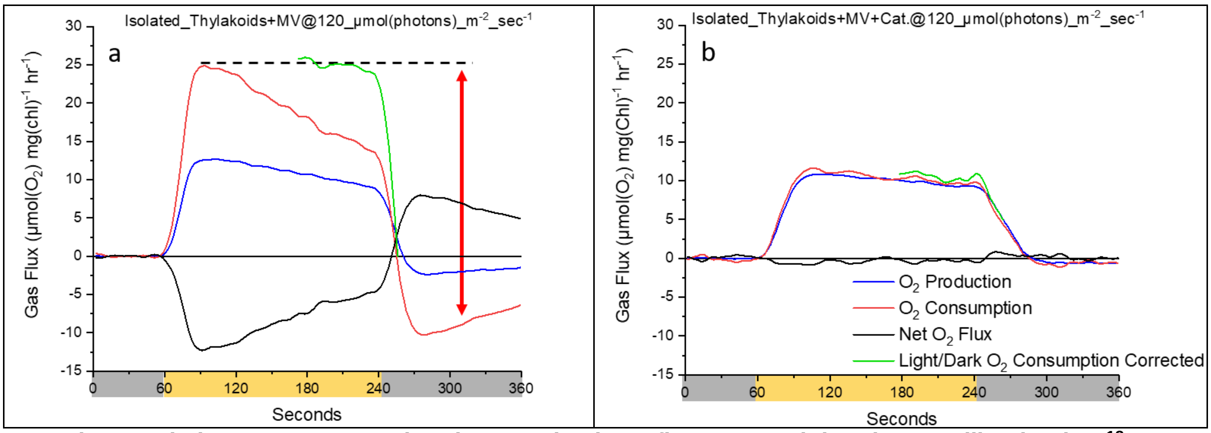

Supplemental Figure S2: Interpreting time resolved gas fluxes, examining the post illumination $^{18}O_2$ burst as further evidence of H_2O_2 accumulation. The data presented in panels a&b are also presented in main Fig. 3, panels c&d (simplified here by exclusion of the standard error). To generate these time resolved gas flux figures, changes in the cuvette's gas concentration were integrated against time (including offsets for isotope dilution, instrument gas consumption etc, which are discussed in detail in methods and for further reading see (Burlacot et al 2020). Examining data in this way provides information pertaining to system dynamics that are otherwise lost when only reporting the maximum rates measured under each condition (as is commonly published). In panel a, we can observe that the rate of O_2 consumption (red line) declines more quickly than the rate of O_2 production (blue line) during illumination (represented by yellow bar on x-axis). This suggests the 1:2 O_2 flux ratio associated with H_2O_2 accumulation does not hold and that H_2O_2 may only accumulate briefly at the initiation of illumination (represented by the yellow bar on x-axis). However, once the light is extinguished at 240 seconds (grey bar in x-axis), the O_2 consumption rate becomes strongly negative. Two important points must be raised with respect to this observation. (i) This trend is absent in panel b, in which catalase precluded the accumulation of H_2O_2. (ii) A 'negative rate' of consumption implies the formation of $^{18}O_2$. The only source of $^{18}O_2$ formation in this system is the decomposition of an accumulated pool of $H_2{}^{18}O_2$. In other words, as H_2O_2 formed with $^{18}O_2$ accumulates into a pool, which then starts to decompose, the background rate of $^{18}O_2$ formation begins to mask the true rate of $^{18}O_2$ consumption. As the H_2O_2 pool size increases with illumination time, the rate of $^{18}O_2$ formation also increases. This leads to the steadily decreasing rate of O_2 consumption when compared to the relatively steady rate of O_2 prodution during illumination. This point is illustrated clearly when we subtract the negative rate of $^{18}O_2$ consumption (from ≈ 240 seconds, ie. negative O_2 consumption) from the illuminated rate $^{18}O_2$ consumption proceeding darkness, represented by the green curves in panels a&b. In panel a, this resulted in a perfect correction to declining rate of 18O2, as highlighted by the black dashed line. In panel b, the small negative rate is a product of the changing concentrations of the two isotope gasses that can lead to a slight error in the dark following a period of illumination. This is a common artefact in MIMS measurements (see Fig 5 in Burlacot et al. 2020). As such, we can use the strong negative rate of O_2 consumption in the pot illuminated samples as evidence for an accumulated pool of H_2O_2.Looking back through all of the data presented in this paper, this phenomenon only occurs in samples in which PSI could be reduced by electrons from PSII, in perfect support of our other lines of evidence.

Reference

Burlacot, A., Burlacot, F., Li-Beisson, Y. & Peltier, G. Membrane Inlet Mass Spectrometry: A Powerful Tool for Algal Research. Front. Plant Sci. (2020) doi:10.3389/fpls.2020.01302

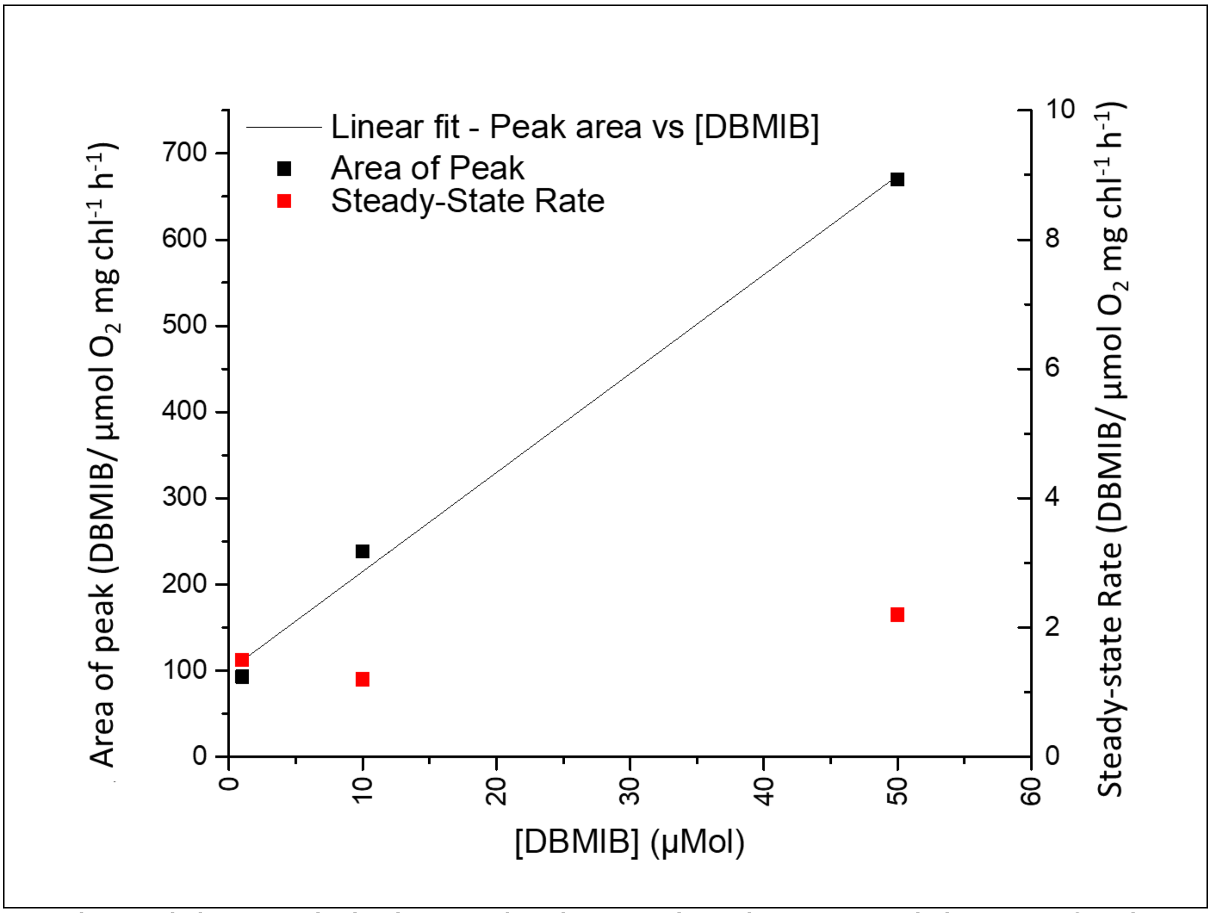

Supplemental Figure S3 Plotting integrated Peak Area and steady-state O_2 Evolution rate as functions of DBMIB Concentration. The comparison highlights that the initial O_2 evolution peak, observed during illumination in thylakoid samples incubated with DBMIB, scaled linearly with increasing concentration between 1 µM and 50 µM, with an adjusted r^2 value of 0.990 (the curve does not pass through zero as O_2 is also produced in the absence of DBMIB). The steady-state rate of O_2 evolution, observed following the peak, did not change very much with changing [DBMIB] concentration over the 50 fold increase in concentration from 1 µM to 50 µM. Only a single replicate of each point was made.

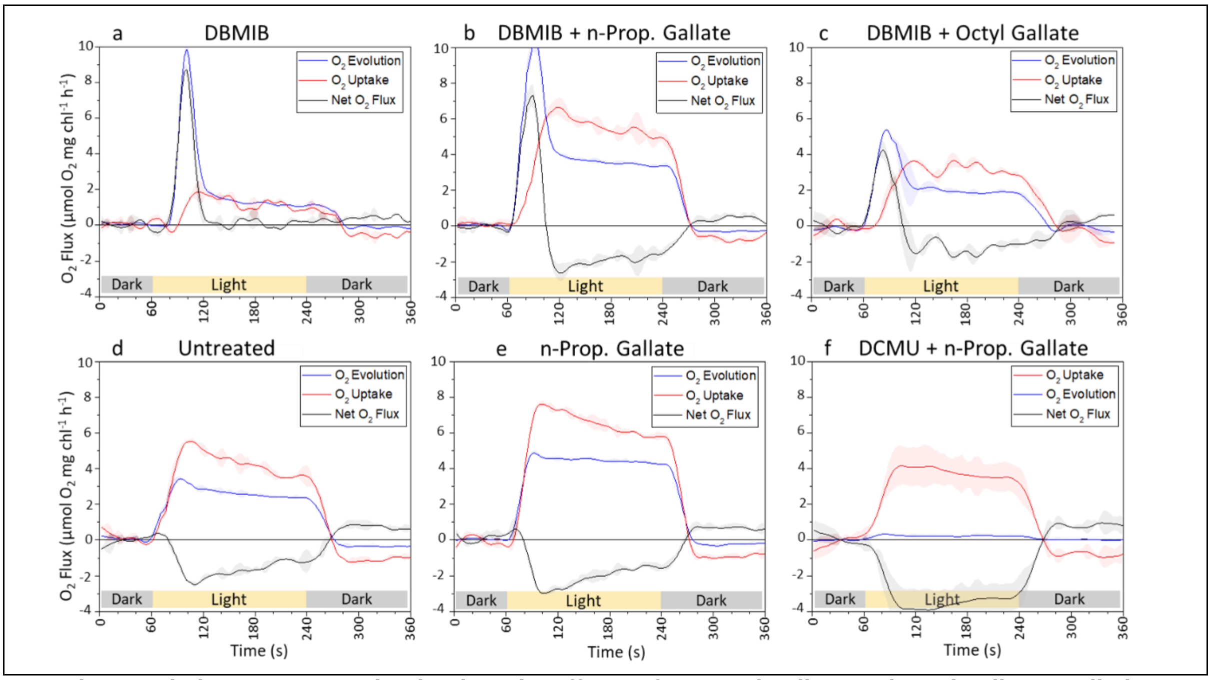

Supplemental Figure S4. Investigation into the effects of n-propyl gallate and octyl gallate applied at 0.5 mM to isolated thylakoid membranes. The same batches of isolated thylakoid membranes used in the main experiments were used to generate these data. Curves were produced by averaging minimum 2 replicates and are drawn including their standard error.

DBMIB treated thylakoid samples exhibit a steady-state of O_2 Evolution and Consumption that implies O_2 reduction is occurring within the thylakoid membrane. We conducted a DBMIB concentration response curve to eliminate this chemical acting as an intermediate for such a reaction, leaving two likely candidates to explain the observation. Either Plastid Terminal Oxidase (PTOX) is active and oxidizing the heavily reduced PQ pool[24] or we have a direct measurement of the proposed $PQH_2/PQH^{-\cdot}$ mechanism able to reduce O_2 to superoxide[43], which our data suggests must be rapidly quenched back to H_2O. In an effort to test the PTOX hypothesis, we applied the commonly used inhibitors of PTOX, *n*-propyl gallate (*n*-pg) and octyl-gallate. Both had similar effects, but we present a focus on the *n*-pg results, including octyl-gallate only in panel c. In panel a, the standard 10 µM DBMIB response to illumination with 120 µmol photons m^{-2} s^{-1} is presented. Panels b and c show the effect of 0.5 mM addition of *n*-pg or octyl gallate to the DBMIB. In contrast to previous reports, the ‘inhibitor’ stimulated rates of both PSII activity and O_2 uptake significantly, suggesting some unusual activity which we cannot explain. To discount DBMIB as being responsible for the effect, we compare untreated thylakoid samples in panel d, with thylakoids treated only with 0,5 mM *n*-pg. Again, the activity of PSII and O_2 consumption was increased significantly. As a final test, in panel f, we inhibited PSII with 10 µM DCMU and again added 0.5 mM *n*-pg. Although PSII was not 100% inhibited, O_2 uptake was stimulated to very high levels. This final result can be explained by the action of the gallates to quench the formation of radicals, especially if chlorophyll triplet states are stimulated by the addition of DCMU. However, we cannot speculate on the cause of the increased PSII activity in all isolated thylakoid. We recommend that thorough controls must be applied to any work in which the gallates have been used to inhibit PTOX, especially testing gas fluxes and not only relying upon fluorescence. As a conclusion to this finding, we cannot discriminate between PTOX function and the action of $PQH_2/PQH^{-\cdot}$ superoxide formation as an explanation for the PSII activity and O_2 uptake we observed in the presence of DBMIB or $HgCl_2$ in our main results.

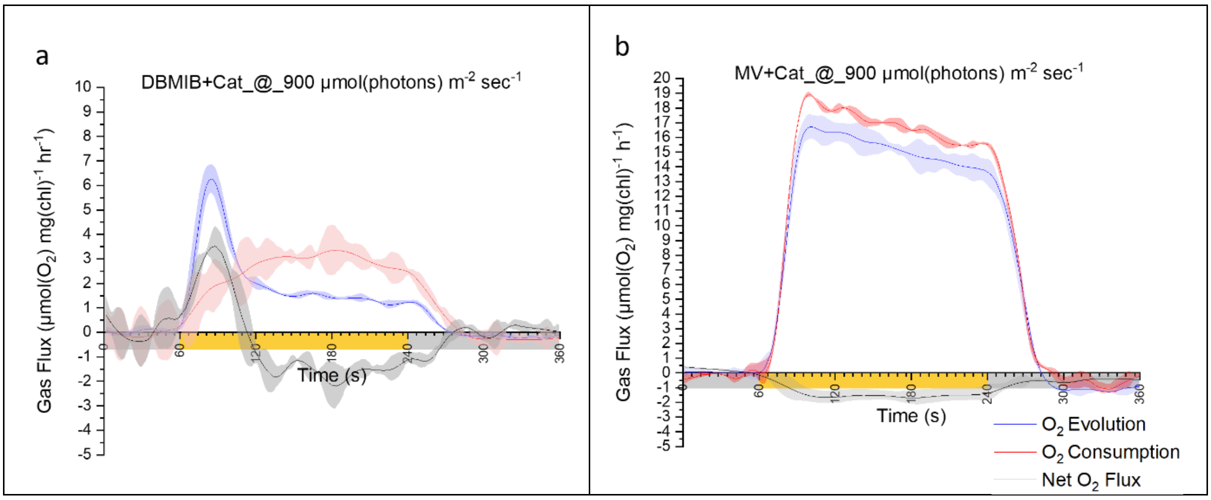

Supplemental Figure S5. Impact of High Light on isolated thylakoid samples treated with catalase was consistent for all measured samples. In the main text it was observed that at Low Light (120 µmol(photons) m^{-2} s^{-1}) all isolated thylakoid samples in which electrons could not reach PSI, or were incubated with catalase, exhibited an O_2 flux ratio of 1:1. This discounted the accumulation of H_2O_2 in these samples. However, at High Light (900 µmol(photons) m^{-2} s^{-1}) the O_2 flux ratio shifted from 1.0 to 0.75 in $HgCl_2$ and DBMIB treated samples (Main text Fig. 4e). In addition, samples incubated with DCMU exhibited a clear light dependent steady state rate of O_2 consumption in the absence of any O_2 production by PSII. This O_2 consumption was consistent with the formation of $^1O_2$ and peroxidation of proteins, membranes and lipids within the sample. We further confirmed that the O_2 consumption in DCMU samples was not a result of H_2O_2 accumulation by incubating them with DCMU+Catalase. In this case we observed no change in the gas flux rates or O_2 stoichiometric ratios (main Fig.4 b,f). The extra O_2 consumption caused by the formation of $^1O_2$ due to the increased excitation pressure in high light treated samples was hypothesized to be the reason for shifting the O_2 flux ratio from 1.0 to 0.75. This was supported by directly subtracting the gas flux rates of DCMU+catalase from those measured with DBMIB and $HgCl_2$, which restored the O_2 flux ratio in those samples to 1.0 (main text Fig 4 g,h). In order to further discount H_2O_2 as a contributor to O_2 consumption in DBMIB samples at High Light, we also added catalase to samples treated with DBMIB. The result, in panel a, is identical to the data presented in main text Fig.4c, which confirms no H_2O_2 was accumulated in DBMIB samples at High Light. Due to the toxicity of Hg_2^+ to catalase, this could not be repeated with $HgCl_2$ treated samples. In order to minimise electron acceptor limitation at High Light, we also measured samples incubated with MV and catalase, panel b. Even in the presence of catalysts for both O_2 reduction at PSI and the decomposition of H_2O_2, forming an unlimited acceptor capacity at PSI in which no H_2O_2 could accumulate, we observed a light dependent rate of O_2 consumption (red line) that was greater than O_2 production (blue line). Interestingly, the magnitude of the difference in O_2 production and O_2 consumption was relatively consistent across all High Light samples treated with catalase (DCMU, DBMIB, MV), despite these systems exhibiting vastly different acceptor side capacities. This suggests that a primary site of $^1O_2$ formation may have been in detached antennae complexes which were not able to transfer energy into reaction centre chlorophylls, regardless of the conditions. In any case, the data suggests that extra O_2 consumption generated through $^1O_2$ formation was the cause of a shift in O_2 flux ratios, justifying the subtraction of DCMU+Catalase
gas fluxes from other samples in main text Fig. 4.

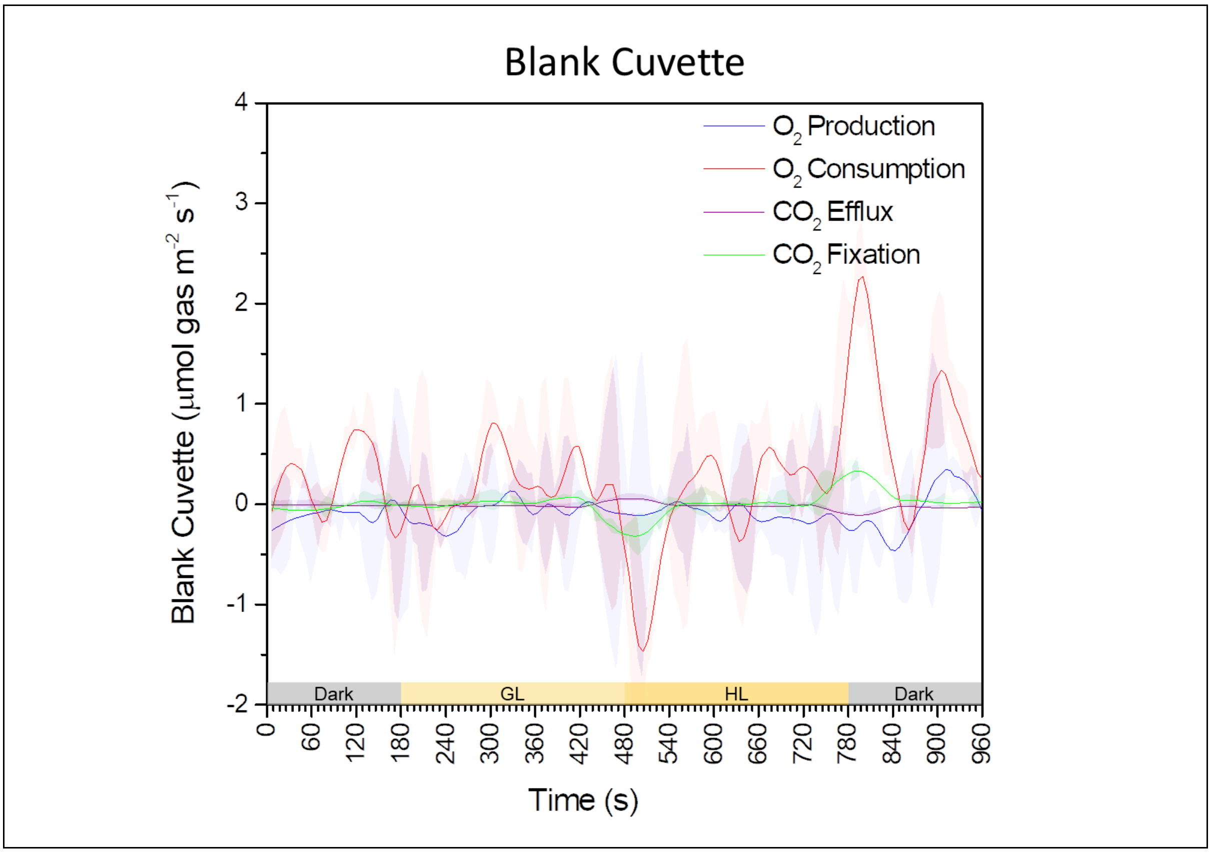

Supplemental Figure S6 Gas fluxes measured in a blank cuvette. Running a procedural blank highlighted that $^{18}O_2$ uptake rates were significantly affected by the switch between GL and HL (apparent O_2 production spike), then HL to Darkness (apparent O_2 Uptake spike). In actual leaf disc measurements these same artefacts were observed, but reached even greater magnitude. Hence, the transitions between light levels have been cut from the main Fig 6 to highlight the gas fluxes on either side of the artefact, which is not uncommon in MIMS measurements and may be due to slight heating on the membrane caused by the light. These curves are the average of two blank runs plotted with the standard deviation.

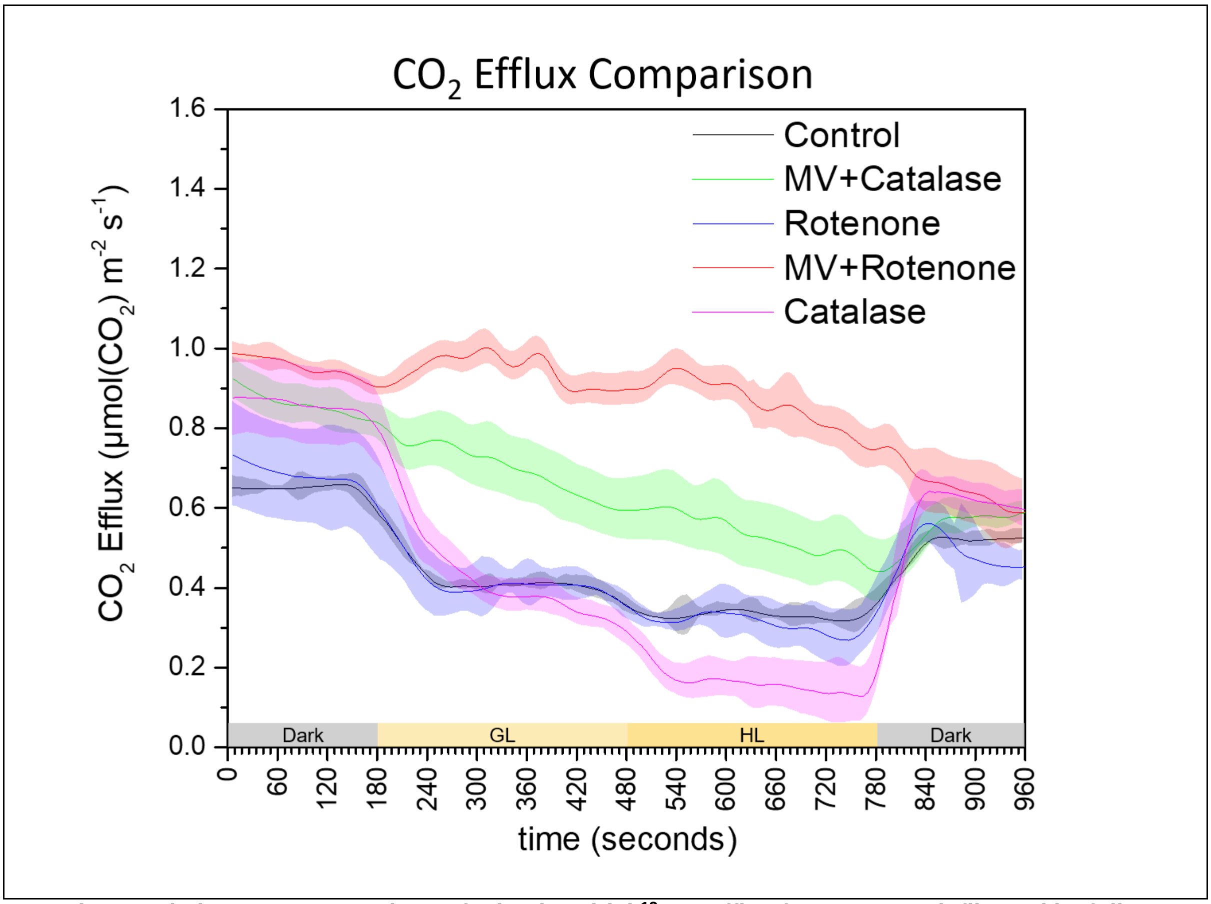

Supplemental Figure S7 Comparison of mitochondrial $^{12}CO_2$ efflux from vacuum infiltrated leaf discs. Rates of $^{12}CO_2$ efflux from leaf discs infiltrated with MV were found to increase significantly during illumination. To test the hypothesis that this may be a result of mitochondrial stimulation caused by H_2O_2 export from the chloroplast, a number of basic experiments were performed. Vacuum, rather than passive infiltration, was used for these follow-up measurements to maximise the ingress of the large catalase molecule. Compared to the control discs infiltrated with water (black line), infiltration with 50 µM rotenone (blue curve, inhibitor of mitochondrial Complex 1) had no effect on either dark or illuminated $^{12}CO_2$ efflux rates. However, disc infiltration with MV plus Rotenone exhibited the same response in both darkness and during illumination as the MV result presented in the main text (Fig. 6c). As such, we tentatively discount Complex 1 from involvement in the observed increase in $^{12}CO_2$ efflux. Discs infiltrated with buffer containing 500 units mL^{-1} catalase (pink curve) exhibited identical trends to the control, although for an unknown reason physiological performance (all measured parameters, including O_2 evolution) were increased at all irradiances. When discs were infiltrated with a combination of 10 µM MV and catalase, the dark respiration rate remained elevated compared to control discs. However, the addition of MV blocked the light dependent $^{12}CO_2$ efflux evident in control, rotenone and catalase samples. As a result, CO_2 efflux remained steady during illumination, similar to DBMIB and DCMU discs in the main figure (Fig. 6d,e). From this observation it can be concluded that MV in the presence of catalase still blocked the majority of CO_2 fixation, by accepting electrons, and was therefore generating H_2O_2. Although catalase didn't effect MV dependent increase in dark respiration, it clearly impaired the large increase in illuminated $^{12}CO_2$ efflux observed in MV treated samples. This supports the hypothesis that MV induced H_2O_2 export was possibly a trigger for increased CO_2 efflux during illumination and therefore, that H_2O_2 export from the chloroplast may be a trigger that stimulates mitochondrial respiration. All curves are the average of minimum 3 replicates, plotted with the standard error.

Table T1. Average Steady-State approximation of rates - µmol(gas) m^{-2} sec^{-1} ± St.Error								
	Control		MV		DBMIB		DCMU	
Irradiance	120	900	120	900	120	900	120	900
O_2 Production	5.1 ± 0.2	9.9 ± 0.6	2.5 ± 0.1	4.0 ± 0.2	0.5 ± 0.1	0.5 ± 0.1	0.1 ± 0.06	0.2 ± 0.2
CO_2 Uptake	4.7 ± 0.3	9.2 ± .6	0.4 ± 0.08	0.5 ± 0.08	0.0 ± 0.0	0.0 ± 0.0	0.1 ± 0.06	0.2 ± 0.2
O_2 Consumption	1.1 ± 0.04	1.2 ± 0.0	4.2 ± 0.2	5.8 ± 0.4	0.9 ± 0.1	1.0 ± 0.1	0.8± 0.1	0.9 ± 0.1
CO_2 Efflux.	0.6 ± 0.04	0.6 ± 0.1	1.1 ± 0.1	0.9 ± 0.1	0.4 ± 0.03	0.4 ± 0.03	0.5 ± 0.04	0.4 ± 0.03
Mehler O_2 Evolution	0.4 ± 0.3	0.7 ± 0.6	2.1 ± 0.2	3.5 ± 0.2	0.5 ± 0.1	0.5 ± 0.1	0.0 ± 0.1	0.0 ± 0.3
Mehler O_2 Consumption	0.4 ± 0.1	0.6 ± 0.4	3.1 ± 0.3	4.9 ± 0.4	0.4 ± 0.1	0.6 ± 0.1	0.4 ± 0.1	0.5 ± 0.2
Mehler O_2 ratio	1.0 ± 0.14	1.1 ± 0.13	0.7 ± 0.05	0.7 ± 0.05	1.1 ± 0.4	0.9 ± 0.3	0 ± 0.3	0 ± 0.3

Supplemental Table S1: Average steady-state approximation of rates of gas exchange in µmol m^{-2} s^{-1} in leaf discs.
